# Pig-to-human lung xenotransplantation: advancing xenogeneic respiratory transplantation and clinical translation

**DOI:** 10.1038/s41392-025-02485-4

**Published:** 2025-11-24

**Authors:** Taeho Kwon, Bong-Seok Song, Kyung Seob Lim

**Affiliations:** 1 Futuristic Animal Resource and Research Center, Korea Research Institute of Bioscience and Biotechnology (KRIBB), Cheongju, Chungbuk Republic of Korea; 2 Advanced Bioconvergence Department, KRIBB School, Korea National University of Science and Technology (UST), Daejeon, Republic of Korea

**Keywords:** Translational research, Biotechnology

In a recently published study in *Nature Medicine*, Jianxing He et al., performed the first successful pig-to-human lung xenotransplantation using a six-gene-edited Bama Xiang pig, in which the lung graft maintained viability and gas exchange for 216 hours in a brain-dead human recipient without evidence of hyperacute rejection.^1^ This achievement demonstrates the potential of xenogeneic lungs as a future solution to the global shortage of donor organs while highlighting the unique immunological and physiological barriers that must be overcome for clinical translation.

The shortage of donor organs remains one of the greatest crises in modern medicine. Lung transplantation, in particular, is limited by the scarcity of suitable donors and the high rate of early graft dysfunction even in allogeneic settings. Xenotransplantation, defined as the transplantation of organs across species, has been envisioned for decades as a possible solution. In recent years, remarkable progress has been made in xenogeneic heart and kidney transplantation, with several high-profile cases demonstrating the short-term feasibility of pig-to-human graft survival.^2^ However, the lung has remained uniquely challenging. Its enormous vascular surface, direct exposure to inhaled pathogens, and vulnerability to ischemia and reperfusion injury combine to create an environment highly susceptible to immune activation and rapid graft loss. Against this background, the successful maintenance of a pig lung in a human recipient for 9 days represents a milestone achievement, providing crucial insights for the future of transplant medicine.

**Fig. 1:**
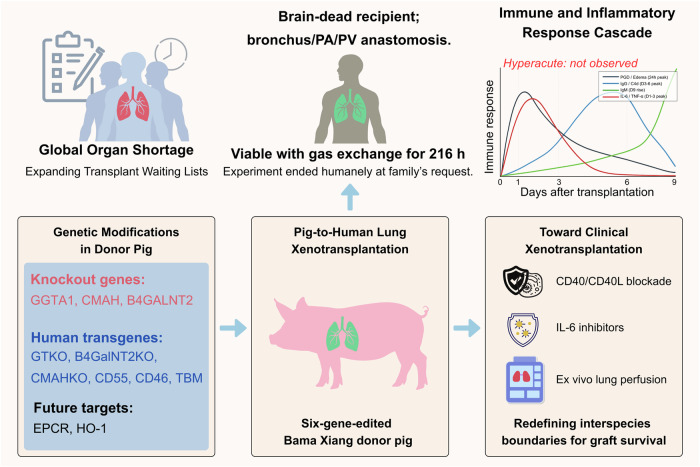
Overview of pig-to-human lung xenotransplantation and translational implications. A six-gene-edited Bama Xiang pig lung was transplanted into a brain-dead human recipient and sustained gas exchange for 216 hours without hyperacute rejection. The figure summarizes the genetic modifications in the donor pig, including knockout of GGTA1, B4GALNT2, and CMAH and insertion of human complement-regulatory genes. Future targets such as EPCR and HO-1 may further enhance graft compatibility. The immune and inflammatory response profile indicates early IgM activation, subsequent IgG deposition, and cytokine storm associated with primary graft dysfunction and antibody-mediated rejection. Key strategies proposed for clinical translation include CD40/CD40L blockade, IL-6 inhibitors, and ex vivo lung perfusion. Together, these integrated approaches highlight the potential of xenogeneic lungs to address the global organ shortage and advance the clinical realization of cross-species transplantation. The figure was created with BioRender.com

As shown in Fig. 1, the donor pig in this study was extensively genetically engineered. Three carbohydrate antigen genes (GGTA1, B4GALNT2, and CMAH) were deleted to eliminate major xenoantigens that drive hyperacute rejection. In addition, three human protective genes (CD46, CD55, and thrombomodulin) were inserted to enhance complement regulation and reduce coagulation abnormalities.^1^ Supplementary sequencing confirmed precise knockouts of the target loci (GGTA1 −1/−1, B4GALNT2 −2/+2, CMAH −1/−1) and stable insertion of the human transgenes.^1^ These edits build on decades of research in nonhuman primates and other preclinical models, where such modifications were shown to reduce immediate graft destruction. The six-gene edited lineage therefore represented one of the most advanced porcine donor platforms yet tested in a human (Fig. 1).

The surgical procedure involved procurement of the left lung from a gene-edited pig under strict biosecure conditions, followed by transplantation into a 39-year-old male brain-dead recipient. Standard techniques were used for anastomosis of the bronchus, pulmonary artery, and pulmonary vein. Postoperative care involved mechanical ventilation, continuous physiological monitoring, serial biopsies, and detailed immunological and microbiological assessments. The observation period extended to 216 hours (9 days), after which the experiment was terminated at the family’s request.^1^

One of the most striking findings was the absence of hyperacute rejection, a catastrophic process that has historically plagued lung xenotransplantation attempts. Hyperacute rejection is typically mediated by preformed natural antibodies binding to pig xenoantigens, activating complement, and causing immediate vascular thrombosis and graft loss. The genetic deletions introduced in this pig appear to have effectively prevented this process. Macroscopic inspection of the graft and histological analyses during the first 2 hours after reperfusion showed intact tissue architecture without evidence of hemorrhage, thrombosis, or widespread necrosis. This result represents a paradigm shift, demonstrating that molecular barriers once considered insurmountable can be overcome.

However, the study also highlighted the formidable challenges that remain. By 24 hours post-transplantation, imaging revealed severe pulmonary edema and consolidation consistent with primary graft dysfunction (PGD). This condition is a major cause of morbidity in clinical lung transplantation and is thought to result from ischemia and reperfusion injury, endothelial activation, and innate immune responses. In this xenogeneic setting, the problem appeared even more pronounced. Histological sections demonstrated infiltration of CD68-positive macrophages and high levels of inflammatory cytokines, with elevated IL-6, IL-8, IL-10, TNF-α, and interferons recorded in serum samples.^1^ This rapid inflammatory cascade underscores the heightened vulnerability of lung xenografts to innate immune damage, a barrier that will require new strategies such as antioxidant therapies, cytokine blockade, or ex vivo conditioning to mitigate.

Antibody-mediated rejection (AbMR) also emerged as a critical barrier. On postoperative days 3 and 6, immunohistochemistry revealed prominent IgG and complement C4d deposition along the alveolar septa, accompanied by tissue injury. In contrast, IgM deposition was minimal early on but became pronounced by day 9, suggesting a delayed humoral response.^1^ Interestingly, these patterns differed from nonhuman primate models, where humoral rejection often develops later and less aggressively. The stronger and earlier activation in humans indicates species-specific immune dynamics, likely driven by the unique antigenic and vascular features of the lung. Despite these challenges, partial recovery of graft function was observed by day 9, with improvements in compliance and gas exchange, suggesting that immune modulation had some stabilizing effect.^3^

The immunosuppressive regimen deployed in this study was among the most comprehensive ever applied in xenotransplantation. It included rabbit anti-thymocyte globulin (rATG) and basiliximab for T cell depletion, rituximab for B cell depletion, eculizumab for complement inhibition, tofacitinib for cytokine suppression, belatacept for costimulation blockade, and maintenance therapy with tacrolimus, mycophenolate mofetil, and tapering steroids.^1^ This combination prevented hyperacute rejection but failed to fully control AbMR. The absence of specific CD40/CD40L pathway blockade, a strategy shown in nonhuman primates to be particularly effective in xenotransplantation, likely contributed to the persistence of antibody responses. Future protocols will need to integrate such targeted approaches, along with agents that block IL-6 or other inflammatory cytokines, to more effectively suppress the unique immune challenges of the lung.

Biosafety was another critical focus of the study. Xenotransplantation carries the risk of transmitting zoonotic infections, particularly porcine endogenous retroviruses (PERVs).^4^ The donor pig used here was derived from a PERV-C–negative lineage, and comprehensive screening confirmed the absence of active PERV infection in both the donor and recipient.^1^ Metagenomic sequencing detected low levels of porcine lymphotropic herpesvirus and bacterial DNA in donor blood, but these were not found in the graft or recipient post-transplantation. Inflammatory markers such as C-reactive protein and procalcitonin declined over time, further suggesting the absence of systemic infection. This meticulous pathogen surveillance provides reassurance regarding biosafety, though it also emphasizes the necessity of rigorous screening protocols in any future clinical application.

The implications of this work are profound. For the first time, a porcine lung has been shown to function in a human for more than a week, providing oxygenation and maintaining compliance despite the immense immunological challenges. This result places the lung alongside the heart and kidney as organs with demonstrated xenotransplantation potential. Yet the study also makes clear that the lung presents a uniquely hostile immunological environment. PGD, rapid AbMR, and intense cytokine storms represent hurdles that are greater than those faced by other organs. The authors propose several strategies to overcome these barriers, including additional genetic modifications such as insertion of EPCR and HO-1 to reduce coagulation and inflammation, ex vivo lung perfusion to condition grafts prior to transplantation, and optimized immunosuppressive regimens that incorporate CD40/CD40L blockade and cytokine-specific inhibitors.^1^

At the same time, the ethical dimensions cannot be overlooked. The use of brain-dead recipients as decedent models was conducted under full ethical approval and national oversight, with explicit family consent obtained specifically for participation in the xenotransplantation study.^1,5^ This consent process was distinct from conventional organ donation, which requires the presence of direct family members. The ethical framework ensured transparent governance under national transplantation authority supervision and provided a responsible precedent for future human decedent research.^1,5^

In conclusion, the study by He et al. represents a watershed moment in xenotransplantation. It demonstrates that with advanced genetic modifications and comprehensive immunosuppression, pig lungs can survive and function in humans, although for a limited period. While major barriers remain, including PGD, antibody-mediated rejection, and infection risk, the path toward clinical lung xenotransplantation is now more clearly defined. Continued innovation in genetic engineering, immunotherapy, biosafety, and organ preservation will be essential. Ultimately, this work brings us closer to a future where xenogeneic lungs may provide life-saving options for patients with end-stage respiratory failure, addressing one of the most urgent unmet needs in global medicine. This achievement unites technological precision with ethical foresight, setting the foundation for clinical xenotransplantation that balances innovation, safety, and social responsibility, and highlights the potential of integrating advanced genetic and immune modulation strategies to achieve durable graft survival and tolerance. The continuous refinement of ethical and biosafety frameworks will be vital for transforming these advances into sustainable therapeutic reality.
